# Alcohol and Cannabis Perceived Descriptive and Injunctive Norms, Personal Use, and Consequences Among 2-Year College Students

**DOI:** 10.3390/bs15030251

**Published:** 2025-02-22

**Authors:** Jennifer C. Duckworth, Kristi M. Morrison, Christine M. Lee

**Affiliations:** 1 Department of Human Development, Washington State University, Pullman, WA 99164, USA; kmgoetz@wsu.edu; 2 Department of Psychiatry and Behavioral Sciences, University of Washington, Seattle, WA 98195, USA; leecm@uw.edu

**Keywords:** alcohol, cannabis, perceived norms, 2-year students, community college students

## Abstract

Two-year college students represent 35% of U.S. undergraduates, yet substance use among them is understudied. Grounded in Social Norms Theory, the present study examined alcohol and cannabis use prevalence and associations between perceived peer use (descriptive norms), approval of use (injunctive norms), and personal use among 2-year students. We also explored whether identification with the reference group or age moderated associations. Data were collected from May through August of 2020 from 1037 2-year college students in Washington State (screening sample) aged 18–29. Of these, 246 participants who reported recent, moderate alcohol and/or cannabis use completed a follow-up survey. Screening survey participants reported past-month alcohol and cannabis use and demographics, while follow-up participants provided data on perceived peer descriptive and injunctive norms and group identification. Screening participants reported drinking an average of 3.32 (*SD* = 7.76) drinks weekly and being high for 8.18 h (*SD* = 20.95). Follow-up participants overestimated peer alcohol and cannabis use. Regression analyses showed perceived descriptive alcohol and cannabis norms were positively associated with personal use, and perceived injunctive alcohol norms were positively related to alcohol-related consequences. Differences by student age were also observed. Findings suggest perceived peer norms are risk factors for substance use behaviors among 2-year college students. Tailored normative feedback interventions may reduce high-risk use in this underserved population.

## 1. Introduction

Nearly 5 million students in the U.S. attend 2-year institutions, representing 35% of the undergraduate population (National Center for Education Statistics, 2023). Two-year institutions are often an important pathway to postsecondary education for underrepresented students (e.g., first-generation college students and students of color) due to low tuition rates, open enrollment policies, and geographic proximity to home (Ma & Baum, 2016). Most 2-year college students are young adults (Mullin, 2012), a developmental period with higher rates of alcohol and cannabis use than any other age group (Patrick et al., 2024). Recent data from Monitoring the Future (MTF) indicates nearly 70% of young adults report drinking alcohol in the past 30 months, 27% reported heavy episodic drinking (HED) in the past 2 weeks (5+ drinks in a row), and 29% used cannabis in the past month (Patrick et al., 2024). Rates of young adult cannabis use have increased in the last ten years, a time when many states have legalized non-medical cannabis use (Patrick et al., 2024). Young adult alcohol and cannabis use has also been linked with negative consequences, including driving while intoxicated, traffic accidents, and unintentional injuries and fatalities (Hingson et al., 2017; White et al., 2011). Identifying risk factors for substance use among young adult 2-year college students is a public health priority; however, research focuses heavily on 4-year college students (Cronce et al., 2022).

Limited research has examined rates of alcohol and cannabis use among 2-year college students, suggesting that 2-year students drink less frequently than 4-year students (Blowers, 2009; Duckworth et al., 2024). For instance, in a recent study, 4-year students consumed an average of seven drinks per week over the past month, compared to an average of four drinks per week among 2-year students (Duckworth et al., 2024). However, high-risk drinking, such as HED, is common among 2-year students, with prevalence rates ranging from 25% to 50% (Cadigan et al., 2019; Velazquez et al., 2011; Wall et al., 2012). Two-year students may also be as likely as 4-year students to report negative alcohol-related consequences (Blowers, 2009; Cremeens-Matthews & Chaney, 2016; Velazquez et al., 2011), suggesting that even though 2-year students may drink less frequently, alcohol use may be associated with greater consequences. Less is known about cannabis use among 2-year students, with some studies reporting 2-year students use more cannabis than 4-year students (Cadigan et al., 2019; Duckworth et al., 2024) and other studies reporting null findings (O’Brien et al., 2018). Research and programing focusing on substance use prevention among 2-year students is needed. The U.S. Department of Education highlights the need to adapt current college substance abuse prevention strategies to meet the needs of 2-year students (American Association of Community Colleges, 2002). Chiauzzi et al. (2011) surveyed 100 administrators, faculty, and health services staff at 100 2-year colleges and found that concerns about student alcohol and cannabis use were common. Researchers called for increased efforts focused on substance use prevention at 2-year institutions, including considering how to effectively deliver social norms programming to 2-year students.

### 1.1. Perceived Social Norms as a Proximal Risk Factor

Perceived peer social norms are a key risk factor for substance use among 4-year students (e.g., Borsari & Carey, 2003; Neighbors et al., 2007). Social Norms Theory (Berkowitz, 2004) posits that perceptions of peer substance use behaviors (descriptive norms) and attitudes (injunctive norms) influence one’s own behaviors and attitudes. For example, studies examining mostly 4-year college students indicate that when students are asked how often they believe their peers drink alcohol (descriptive norms) or how much they think their peers approve of alcohol-related behaviors such as HED (injunctive norms), students often overestimate both peer use and peer approval of use, and these overestimations are in turn associated with increased personal use (Baer et al., 1991; Borsari & Carey, 2003). While most research has focused on alcohol use—historically more prevalent on college campuses (Borsari & Carey, 2003)—the increasing legalization and use of cannabis among young adults have led to greater study of perceived norms in cannabis consumption (Napper et al., 2016; Patrick et al., 2024). Like alcohol, 4-year students often overestimate peer cannabis use and approval, which is linked to higher personal use (Kilmer et al., 2006; Napper et al., 2016). These misperceptions are reinforced through social learning mechanisms where exposure to substance use within peer networks normalizes the behavior and increases its acceptability (Bandura, 1986). The college experience, with exposure to new social groups, reduced parental supervision, and increased autonomy, heightens the impact of perceived peer norms (Borsari & Carey, 2001). Additionally, alcohol and cannabis use are often integrated into campus social life, increasing students’ susceptibility to peer influence (Bandura, 1986). Based on these findings, effective stand-alone and multi-component social norms interventions have effectively reduced high-risk behaviors among 4-year students (e.g., Lewis et al., 2014; Neighbors et al., 2015; Riggs et al., 2018), particularly alcohol use (Dotson et al., 2015; Moreira et al., 2009). Personalized normative feedback, which corrects misperceptions of peer alcohol norms, has been shown to decrease personal use (Larimer et al., 2007; Lewis & Neighbors, 2006; Neighbors et al., 2004) and is listed as a higher effectiveness individual-level strategy in the NIAAA CollegeAIM matrix (Cronce et al., 2018). Fewer studies have tested normative feedback for cannabis use, with mixed results (Elliott & Carey, 2012; Lee et al., 2010; Riggs et al., 2018).

While social norms interventions have been effective at reducing substance use among 4-year students, important differences exist between 2- and 4-year students. Two-year students are much more diverse than 4-year students in terms of demographics (e.g., age, socioeconomic status; Mullin, 2012), and 2-year students are more likely to be part-time students, take online courses, live off campus (Bambara et al., 2009; Eren & Keeton, 2015; Wall et al., 2012), and have adopted adult social roles (e.g., parents, full-time employees; National Center for Education Statistics, 2023). These differences could impact how substance use norms operate for 2-year students. A recent study found that higher perceived descriptive norms were related to increased personal use among 2- and 4-year college students (Duckworth et al., 2024). Two-year status did not moderate associations, suggesting the relationship between perceived descriptive norms and personal use function similarly for 2- and 4-year students. However, researchers did not examine if perceived injunctive norms were related to personal use, and the study sample included a relatively low-risk sample (i.e., students who reported alcohol use at least once in the past year). Examining associations among a higher-risk sample of 2-year students is important as they would be the target audience for personalized normative feedback interventions. Researchers also did not examine the role of the reference group for 2-year students.

### 1.2. The Reference Group

The reference group plays a pivotal role in personalized normative feedback interventions by providing a comparison framework for participants (Borsari & Carey, 2003). Social Comparison Theory (Festinger, 1954) and Social Impact Theory (Latané, 1981) suggest more proximal reference groups will have a greater influence than more distal comparison groups, and empirical research with 4-year students suggests that when an individual identifies more strongly with the reference group, the intervention tends to be more effective (Neighbors et al., 2010; Reed et al., 2007). Most research among college students uses “the typical college student” or gender-specific versions as the peer reference group (Lewis & Neighbors, 2006; Neighbors et al., 2010). However, no studies to our knowledge have examined if the proximity to the reference group impacts associations between perceived peer substance use norms and personal substance use among 2-year college students.

### 1.3. The Present Study

Given that Washington State was one of the first states to legalize non-medical cannabis use for individuals aged 21 and older and that 2-year colleges are integral to Washington State’s higher education system, serving over 250,000 students annually across 34 community and technical colleges (Washington State Board for Community and Technical Colleges [WSBCTC], 2023), Washington State is an ideal setting to examine alcohol and cannabis use norms. Two-year institutions in Washington State are also marked by significant diversity (e.g., a student body that includes first-generation students, individuals from low-income backgrounds, and those balancing education with work and family responsibilities; Washington State Board for Community and Technical Colleges [WSBCTC], 2023) and the state’s progressive policies (e.g., the Washington College Grant) enhance access and affordability for students from varied socioeconomic backgrounds (Washington Student Achievement Council [WSAC], 2023).

The present study had three aims. Aim 1 was to determine prevalence rates of alcohol and cannabis use among 2-year college students in Washington State, where alcohol and cannabis use are legal for individuals aged 21 and older. We examined a large screening sample of 2-year students, and given limited research, we considered this aim to be exploratory. For Aim 2, we examined a follow-up sample of 2-year college students who reported recent, moderate alcohol and/or cannabis use to investigate if they overestimated peer alcohol and cannabis use as reported in the large screening sample. We hypothesized that 2-year students would overestimate peer use. For Aim 3, we examined the follow-up sample of 2-year students who reported recent, moderate alcohol and/or cannabis use to determine (a) if perceived descriptive and injunctive alcohol and cannabis norms were associated with greater personal use and related consequences, and (b) whether identification with the reference group or student age moderated associations. We hypothesized that perceived descriptive and injunctive norms would be positively linked with personal use and consequences. We also hypothesized that greater identification will be related to stronger associations between perceived peer alcohol and cannabis norms and personal alcohol and cannabis use. We considered tests of moderation by age to be exploratory.

## 2. Materials and Methods

### 2.1. Participants and Procedures

Two-year college students in Washington State were recruited into this study through social media and online advertising, targeted emails to student groups and faculty at local 2-year colleges, and fliers posted at local 2-year colleges. Interested students completed a brief, confidential online screening survey from May through August of 2020, during the COVID-19 pandemic. A total of 3411 individuals completed the screening survey. Of those, 55% were ineligible because they were not 2-year college students (*n* = 1867), 234 did not live in Washington State, 36 individuals did not give consent, and 25 were not between the ages of 18 and 29. Thus, 1249 students were eligible for the screening survey, and those with non-missing data on variables of interest were included in the present study (*n* = 1037). Students did not receive payment for participating in the screening survey, which included 45 questions and took participants about 20 min to complete. A subset of students who reported recent, moderate use (i.e., HED in the past month and/or cannabis use at least three times in the past month) were invited to participate in a follow-up survey. A total of 245 students who reported recent, moderate alcohol (*n* = 204) and/or cannabis use (*n* = 169) completed follow-up online surveys within one month of completing the screening survey. Students received a USD 20 gift card for participating in the follow-up survey, which consisted of 80 questions and took participants about 30 min to complete. All study procedures were approved by the university’s Institutional Review Board (IRB).

### 2.2. Measures

#### 2.2.1. Screening Survey

Demographic Characteristics. Participants reported their biological sex at birth (0 = male; 1 = female), race, ethnicity, and age in the initial screening survey. Given age differences in both perceived peer substance use norms and personal substance use (Oh et al., 2022; Patrick et al., 2024), for moderation analyses, we dummy coded student age as 18–20 and 26–29 with 21–25 comprising the reference group.

Alcohol Use. Participants completed the Daily Drinking Questionnaire (DDQ; Collins et al., 1985), where they reported how many alcoholic drinks they consumed each day during a typical week during the past month. Total drinks consumed in a typical week were calculated from the sum of each day. Participants then reported how many times they had engaged in HED in the past month (4/5 or more drinks in a single occasion for females/males, respectively), based on sex at birth. Responses were coded into a binary variable where 1 = engaged in HED in the past month and 0 = did not engage in HED in the past month.

Cannabis Use. Participants reported how many hours they were high each day in a typical week during the past month using the Daily Marijuana Questionnaire (DMQ; Lee et al., 2013). Total hours high in a typical week were calculated from the sum of each day.

#### 2.2.2. Follow-Up Survey

Alcohol Outcomes and Perceived Peer Alcohol Norms. Participants in the follow-up survey also reported past-month drinks per week using the DDQ (Collins et al., 1985; summed) and HED with responses recoded into a binary variable (0 = No HED; 1 = HED). Participants indicated whether they experienced 24 consequences as a result of drinking during the past month (0 = no, 1 = yes) using the Brief Young Adult Alcohol Consequences Questionnaire (BYAACQ; Kahler et al., 2005), with items summed. For perceived descriptive alcohol norms, participants reported how many drinks they thought the typical community college student of the same age and gender drank each day of a typical week in the past month with the number of drinks summed, and also how many times they thought the typical community college student of the same age and gender engaged in HED in the past month. Similar to personal HED, response options were recoded into a binary variable. For perceived injunctive alcohol norms, participants were asked how much they thought the typical community college student of the same age and gender approved of drinking alcohol every weekend, drinking alcohol daily, driving a car after drinking, and drinking enough alcohol to pass out (Baer, 1994; LaBrie et al., 2010). Items were rated on a 7-point Likert scale (1 = strongly disapprove; 7 = strongly approve; α = 0.74).

Cannabis Outcomes and Perceived Peer Cannabis Norms. Participants reported on how many days they used cannabis in the past 30 days and total hours high per day (summed) using the DMQ (Lee et al., 2013). Participants also reported a frequency of 26 consequences while using cannabis during the past 30 days using the Marijuana Consequences Checklist (Lee et al., 2021), rated on a 5-point scale including (0), 1–2 times (1), 3–5 times (2), 6–10 times (3), and more than 10 times (4), with item scores summed. For perceived descriptive cannabis norms, participants reported how many hours they thought the typical community college student of the same age and gender was high each day in a typical week during the past month (summed). For perceived injunctive cannabis norms, participants reported how much they thought the typical community college student of the same age and gender approved of trying marijuana once or twice, using marijuana occasionally, and using marijuana regularly (Napper et al., 2016) on a 7-point Likert scale (1 = strongly disapprove; 7 = strongly approve; α = 0.88).

Identification with the Reference Group. Adapted from Phua (2013), participants were asked how similar they felt they were to the typical community college student of the same age and gender. Items were rated on a 5-point scale including 0 (not at all similar), 1 (slightly similar), 2 (somewhat similar), 3 (moderately similar), and 4 (extremely similar).

### 2.3. Analytic Strategy

Analyses were conducted using SAS 9.4 software (SAS, 2014). To address outliers on alcohol and cannabis outcomes (i.e., drinks per week, alcohol consequences, hours high per week, and cannabis consequences), we adjusted outliers to one unit greater than 3.29 standard deviations above the mean, following procedures by Tabachnick and Fidell (2019).

- Aim 1: To determine prevalence rates of alcohol and cannabis use among 2-year college students, we descriptively examined use among the large screening sample of 2-year students.
- Aim 2: To determine if 2-year college students who reported recent, moderate use overestimated peer alcohol and cannabis use, we conducted *t*-tests to examine differences in (1) drinks in a typical week as reported in the large, screening sample and perceived peer drinks per week as reported in the follow-up sample of those who reported recent, moderate use and (2) hours high in a typical week as reported in the screening sample and perceived peer hours high per week as reported in the follow-up sample. We also conducted chi-square tests to examine differences in the percent of 2-year students who engaged in past-month HED as reported in the screening sample and the perceived percent of 2-year students who engaged in HED in the past month as reported in the follow-up sample.
- Aim 3a: Using the follow-up sample of 2-year students who reported recent, moderate use and controlling for participant age and sex (0 = male, 1 = female), we examined associations between (1) perceived peer alcohol and cannabis use, and personal use and related consequences, and between (2) perceived peer approval of alcohol and cannabis use, and personal use and related consequences. Because all substance use outcomes were positively skewed, negative binomial regression models were used, and results are reported as rate ratios (RR), which represent the degree to which substance use outcomes change with a one-unit increase in the predictor.
- Aim 3b: We examined if identification with the reference group or student age moderated associations between perceived peer use and approval of use with personal use.

## 3. Results

In the initial screening survey, 1037 participants had data on variables of interest (83% of those that were eligible for the screening survey) and were, on average, 21.36 years old (SD = 2.99) and 63% female. About one-fifth (19.8%) identified as Hispanic or Latinx, 1.6% as American Indian or Alaska Native, 13.4% as Asian, 7% as Asian American, 7% as Black or African American, 1.7% as Native Hawaiian or Pacific Islander, 55.8% as White or Caucasian, 9.3% as multiracial, and 4.1% as other. See Table 1 for additional descriptive statistics.

In the follow-up survey with students who reported recent, moderate use, 246 students had data on variables of interest (94% of those that completed the follow-up survey), had a mean age of 21.69 years old (*SD* = 2.87), and 55.69% were female. Over one-quarter (28.76%) of participants identified as Hispanic or Latinx. Nearly 3% of students (2.97) identified as American Indian or Alaska Native, 7.63% as Asian, 2.97% as Asian American, 4.66% as Black or African American, 3.39% as Native Hawaiian or Pacific Islander, 65.68% as White or Caucasian, 8.05% as multiracial, and 4.66% as other.

### 3.1. Aim 1: Prevalence of Alcohol and Cannabis Use Among 2-Year College Students

Descriptive statistics on the larger screening sample of 2-year students in Washington State are reported in Table 1. Among the screening sample of 2-year students, 52.9% of students reported drinking alcohol in the past month, the mean number of drinks consumed in a typical week was 3.32 (*SD* = 7.76), and about one-third (33.1%) reported engaging in HED on at least one occasion in the past month. Further, 36.2% reported using cannabis in the past month, and the mean hours of being high in a typical week equated to 8.18 (*SD* = 20.95).

### 3.2. Aim 2: Differences in Perceived Peer Alcohol and Cannabis Use and Personal Use

**Figure 1:**
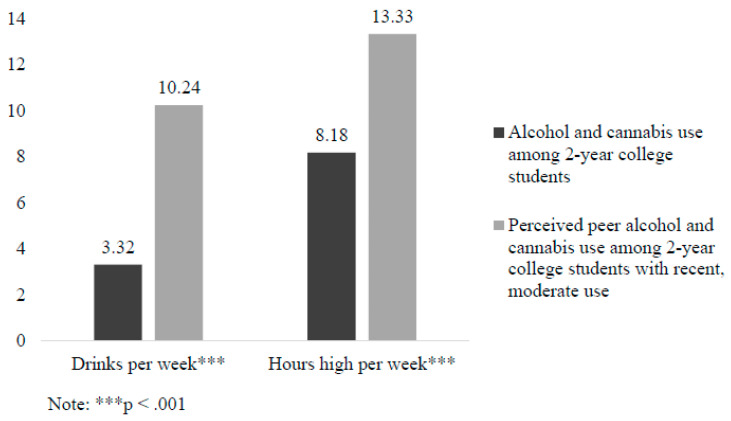
Differences in alcohol and cannabis use among 2-year college students and perceived peer alcohol and cannabis use among 2-year college students who reported recent use.

**Figure 2:**
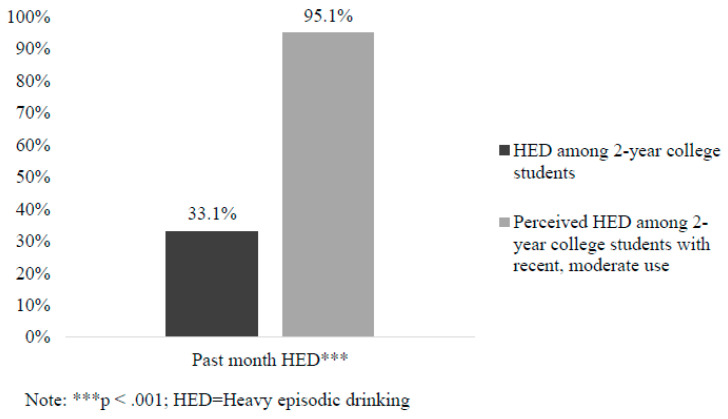
Differences in percent of 2-year college students who engaged in HED and perceived percent of peer HED among 2-year college students with recent use.

Descriptive statistics on the follow-up sample of 2-year students in Washington State who reported recent, moderate alcohol or cannabis use are reported in Table 1. Results for *t*-tests of differences in perceived peer alcohol and cannabis use and personal use are presented in Figure 1 and Figure 2. Significant differences were found such that 2-year students who reported recent, moderate use perceived that their peers consumed more drinks in a typical week (*M* = 10.24, *SD* = 7.21) than were reported in the screening sample (*M* = 3.32, *SD* = 7.76; *t* = 12., *p* < 0.001). For HED, 2-year students who reported recent, moderate use perceived that over 95% of their peers engaged in HED in the past month, whereas only 33% of 2-year students in the screening sample reported actually engaging in HED in the past month (*X^2^* (1, *N* = 1261) = 306.74; *p* < 0.001). For cannabis use, 2-year students who reported recent, moderate use perceived that their peers spent significantly more hours high in a typical week (*M* = 13.33, *SD* = 12.74) than those in the screening sample reported (*M* = 8.18, *SD* = 20.95; *t* = 3.63, *p* < 0.001).

### 3.3. Aim 3: Associations Among Perceived Peer Descriptive and Injunctive Alcohol and Cannabis Norms, Personal Use, and Related Consequences

Alcohol Outcomes. Results for associations between perceived peer alcohol norms and personal alcohol use outcomes among 2-year students who reported recent, moderate use are reported in Table 2. For perceived descriptive norms, controlling for participant age and sex, greater perceived peer drinks per week were positively associated with increased personal drinks per week [RR (95%CI) = 1.03 (1.01–1.05), *p* = 0.005], and perceived peer HED was positively linked with personal HED [OR (95%CI) = 15.93 (1.99–127.34), *p* = 0.009]. For perceived injunctive norms, greater perceived peer approval of alcohol use was associated with increased personal alcohol-related consequences [RR (95%CI) = 1.21 (1.04–1.41), *p* = 0.015], but not personal drinks per week or HED. In moderation analyses (Aim 3b), identification with the reference group did not moderate associations for any of the alcohol use outcomes. However, student age did moderate associations such that relative to students aged 21–25, the association between perceived peer drinks per week (descriptive norms) and personal drinks per week was less pronounced for students aged 26–29 (*β* = −0.07, *p* = 0.025), and the association between greater perceived peer approval of alcohol use (injunctive norms) and personal alcohol-related consequences was less pronounced for students aged 26–29 (*β* = −0.63, *p* = 0.004).

Cannabis Outcomes. Results for associations between perceived peer cannabis norms and personal cannabis use outcomes are reported in Table 3. Controlling for age and sex, greater perceived peer hours high per week was associated with greater personal hours high per week [RR (95%CI) = 1.02 (1–1.04), *p* = 0.03]. Perceived peer approval of cannabis was not significantly associated with cannabis use outcomes. In moderation analyses, neither identification with the reference group nor age moderated associations for the cannabis use outcomes; however, student age moderated associations relative to students aged 21–25 and the association between greater perceived peer approval of cannabis use (injunctive norms) and personal cannabis-related consequences was less pronounced for students aged 18–20 (*β* = −0.63, *p* = 0.049).

## 4. Discussion

Few studies report prevalence rates of alcohol and cannabis use among 2-year college students, and the prevalence rates that are reported are not derived from national samples (e.g., Cadigan et al., 2019; Wall et al., 2012). Compared to national estimates from MTF (Patrick et al., 2024), a smaller proportion of 2-year students in our screening sample reported past-month alcohol use (52.9%) compared to 2- and 4-year college students in MTF (62.5%), but prevalence of HED was slightly higher in our sample (33.1% in our sample; 27.7% in MTF). For cannabis use, more students in our screening sample reported past-month cannabis use (36.2%) compared to 2- and 4-year students in the MTF study (22.1%). Recent work (Cadigan et al., 2019; Duckworth et al., 2024) suggests that 2-year students may be at increased risk for cannabis use relative to 4-year students. However, these samples were drawn from Washington State, where nonmedical cannabis use is legal for adults over the age of 21, which may confound findings. For instance, a recent study found that cannabis use among female 2-year college students who use alcohol had differential findings for those living in states without non-medical cannabis legalization (Binger et al., 2023). More research is needed to understand differences in cannabis use among 2-year college students living in states with different cannabis use legislation.

Consistent with prior research examining descriptive peer norms among 4-year college students (e.g., Baer et al., 1991; Borsari & Carey, 2003; Kilmer et al., 2006), students in the follow-up sample (i.e., students who reported recent, moderate use) overestimated how much other 2-year students of the same gender drank alcohol, engaged in HED, and used cannabis. For example, screening sample participants reported drinking three drinks and being high for 8 h per week on average, whereas follow-up participants perceived that their peers were drinking ten drinks and were high for 13 h per week on average. As hypothesized, results also indicated overestimations were positively related to personal use and consequences. Specifically, for descriptive norms, higher perceived peer alcohol use was related to greater personal drinking and HED, and higher perceived peer cannabis use was related to greater personal cannabis use. Findings are consistent with Social Norms Theory (Berkowitz, 2004) and suggest that interventions aimed at decreasing misperceptions of peer alcohol and cannabis use may be effective in decreasing personal use.

For injunctive norms, higher perceived peer approval of alcohol use was related to more personal alcohol-related consequences, while perceived peer approval of cannabis use was unrelated to personal use or consequences. The finding that perceived injunctive norms were not related to personal alcohol or cannabis use was contrary to our hypotheses and is inconsistent with Social Norms Theory (Berkowitz, 2004). However, this finding is consistent with prior research among 4-year student samples that descriptive norms tend to be more strongly associated with substance use behaviors than injunctive norms (Borsari & Carey, 2003; Zou & Savani, 2019), suggesting that young adults’ perceptions of their peers’ behaviors may influence their own actions more than their perceptions of their peers’ attitudes. Additionally, the finding that injunctive norms were related to alcohol-related consequences but not cannabis-related consequences may stem from how injunctive norms were assessed: participants evaluated perceived peer approval of high-risk alcohol behaviors (e.g., daily drinking and driving after drinking) but lower-risk cannabis behaviors (e.g., trying cannabis once or twice and using cannabis occasionally). Findings for injunctive alcohol norms may be due to the long-standing cultural normalization of alcohol in social settings, particularly among young adults. Alcohol use is often portrayed as a typical part of college life, making peer attitudes more influential in shaping individual behaviors (Borsari & Carey, 2001). The social nature of drinking, coupled with its integration into social activities, may heighten the impact of peer norms in encouraging alcohol use (Neighbors et al., 2007). In contrast, cannabis use, though legal in Washington State, may not exhibit the same degree of peer influence. Cannabis use can still carry some stigma or may be viewed as more private and individualized compared to alcohol. Factors such as medical use or personal coping strategies might play a larger role in cannabis consumption, making it less dependent on peer approval (Kilmer et al., 2006).

Contrary to Social Comparison Theory (Festinger, 1954) and Social Impact Theory (Latané, 1981) and inconsistent with prior research of 4-year students and alcohol use (e.g., Reed et al., 2007), identification with the reference group did not moderate associations between perceived peer norms and personal use and consequences. Social Comparison Theory (Festinger, 1954) and Social Impact Theory (Latané, 1981) suggest that more proximal reference groups exert a stronger influence than more distal reference groups. Research with 4-year college students supports this notion, demonstrating that interventions are generally more effective when individuals feel a stronger connection to the reference group (Neighbors et al., 2010; Reed et al., 2007). Given that 2-year students are a much more diverse group than 4-year students (Snyder et al., 2019; Velazquez et al., 2011), it may be that the typical same-gender community college student is not the best reference group for 2-year college students. Indeed, follow-up participants in this study only somewhat identified with the typical same-gender community college students. Future research could explore if other reference groups are more salient for community college students, such as typical same-gender, same-age young adults.

Finally, student age did moderate associations between perceived peer norms and personal use and consequences, which was an exploratory research question. For alcohol use, associations between perceived peer drinks per week (descriptive norms) and personal drinks per week were less pronounced for older students relative to students aged 21–25, as were associations between greater perceived peer approval of alcohol use (injunctive norms) and personal alcohol-related consequences. These findings are not surprising given developmental, social, and contextual differences between young adults in their late 20s and young adults in their early-to-mid 20s. For instance, according to Erikson’s Psychosocial Theory of Development, younger students are often still in the late adolescence stage of development and may be more susceptible to peer influence as they navigate the Identity vs. Role Confusion stage, where social approval and experimentation with roles are central (Orenstein & Lewis, 2022). In contrast, older students are often transitioning into Erikson’s Intimacy vs. Isolation stage and may be less focused on peer approval and more influenced by life roles, such as work, relationships, and future aspirations (Orenstein & Lewis, 2022). Older students are also more likely to have a realistic understanding of peer behavior, weakening the influence of exaggerated perceptions of drinking norms (Staff et al., 2010). Our findings, coupled with this developmental trajectory, underscore the fact that interventions addressing perceived norms may be most effective for younger college students.

### Limitations and Future Directions

This study should be considered in light of important limitations. Firstly, this study used a convenience sample, which may limit the generalizability of our findings. This is especially important for Aim 1, as our reporting of prevalence rates of alcohol and cannabis use is specific to a convenience sample of 2-year college students in Washington State. Relatedly, this study was conducted in a state where nonmedical cannabis use is legal for adults 21 and older. Both the prevalence of cannabis use and perceptions of peer cannabis use may be different for young adults who live in states where nonmedical cannabis use is illegal. There were also differences in how injunctive norms were assessed. Specifically, participants evaluated perceived peer approval of high-risk alcohol behaviors (e.g., daily drinking) but lower-risk cannabis behaviors (e.g., trying cannabis once or twice). An additional limitation is that this study was conducted in 2020 during the COVID-19 pandemic, during which college student alcohol and cannabis use generally declined (Hussong et al., 2023; Patrick et al., 2024). There is also some evidence to suggest that young adults’ perceptions of peer use may have increased during the pandemic (Graupensperger et al., 2021). Therefore, the results of the current study may be biased by the effects of the COVID-19 pandemic. Finally, all measures were self-reported, which could introduce bias, and causality cannot be determined as these data were analyzed cross-sectionally.

Future research should explore whether reference groups beyond the “typical same-gender community college student” are more salient for 2-year college students. Future studies could explore whether perceptions of alcohol and cannabis use by peers of the same gender and age group outside the college context—such as young adults in general—play a more significant role in shaping personal behaviors. Future studies should also investigate whether the observed associations differ for young adults residing in states where nonmedical cannabis use remains illegal, as legal context may moderate the relationship between perceived peer norms and personal substance use. These directions can enhance our understanding of the nuanced factors shaping substance use among 2-year college students and inform the development of effective prevention and intervention strategies.

## 5. Conclusions

Two-year college students represent a large, growing, and understudied population in the United States (Juszkiewicz, 2017). Although the majority of 2-year college students are young adults, a developmental period associated with increased risk for substance use and related consequences (Patrick et al., 2024; Schulenberg & Maggs, 2002), little is known about substance use and risk factors for substance use among 2-year students. The current study examined prevalence rates of alcohol and cannabis use among 2-year students and associations among perceived descriptive and injunctive alcohol and cannabis norms and personal alcohol and cannabis use outcomes. Over half of the screening sample reported recent alcohol use, one-third engaged in HED, and over a third reported cannabis use. However, students’ perceptions of peer behavior among a subsample who reported recent moderate use exceeded actual reported usage among the screening sample. These inflated perceptions were linked with increased personal substance use and alcohol-related consequences, particularly among younger students. Findings underscore the importance of addressing misperceptions of peer norms in prevention and intervention efforts targeting substance use among 2-year college students and present promising avenues for applied substance use prevention research. For example, findings could inform the development of a campus-based prevention program using a social norms approach (DeJong, 2010). This could include the development and pilot testing of personalized feedback interventions targeting reductions in alcohol and cannabis use among 2-year college students (Lewis & Neighbors, 2006). Such programming could leverage insights from perceived peer social norms to tailor messages that challenge overestimations of peer use and promote healthier behaviors.

## Figures and Tables

**Table 1:** Descriptive statistics.

	Screening Sample *n* = 1037	Follow-Up Sample *n* = 246
	*M* (*SD*) or *n* (%)	*M* (*SD*) or *n* (%)
Age	21.36 (2.99)	21.69 (2.87)
18–20	524 (50.50)	99 (40.91)
21–25	395 (38.10)	118 (48.76
26–29	118 (11.40)	25 (10.33)
Female	650 (62.70)	137 (55.69)
Race		
American Indian/Alaska Native	16 (1.60)	7 (2.97)
Asian	134 (13.40)	18 (7.63)
Asian American	70 (7.00)	7 (2.97)
Black/African American	70 (7.00)	11 (4.66)
Native Hawaiian/Pacific Islander	17 (1.70)	8 (3.39)
White/Caucasian	556 (55.80)	155 (65.68)
Multiracial	93 (9.30)	19 (8.05)
Other	41 (4.10)	11 (4.66)
Hispanic ethnicity	185 (19.80)	65 (28.76)
Alcohol outcomes		
Alcohol use in past month	549 (52.90)	204 (83.30)
Drinks per week	3.31 (7.76)	5.5 (6.02)
HED in past month	336 (33.10)	143 (58.13)
Alcohol-related consequences ^a^	--	5 (5.35)
Perceived peer alcohol use		
Perceived peer drinks per week	--	10.24 (7.21)
Perceived peer HED in past month	--	233 (95.10)
Perceived peer approval of alcohol ^b^	--	3.43 (1.11)
Cannabis outcomes		
Cannabis use in past month	369 (36.20)	169 (68.98)
Days using cannabis	--	10.13 (11.66)
Hours high per week	8.18 (20.95)	14.2 (20.25)
Cannabis-related consequences ^c^	--	13.9 (15.23)
Perceived peer cannabis use		
Perceived peer hours high per week	--	13.33 (12.74)
Perceived peer approval of cannabis ^b^	--	5.07 (1.14)
Identification with reference group ^d^	--	1.84 (1.02)

^a^ Alcohol consequences represent the number of consequences endorsed in the past month. ^b^ Response options ranged from 1 (strongly disapprove) to 7 (strongly disapprove). ^c^ Cannabis consequences are a scale that represents how often participants experienced cannabis-related consequences in the past month. ^d^ Response options ranged from 0 (not at all similar) to 5 (extremely similar).

**Table 2:** Rate ratios or odds ratios (and 95% CIs) for associations among typical alcoholic drinks per week, heavy episodic drinking, alcohol-related consequences, and descriptive and injunctive alcohol norms among 2-year college students with recent moderate use.

	Drinks per Week		Heavy Episodic Drinking (HED)		Alcohol-Related Consequences	
	Rate Ratio	(95% CI)	Odds Ratio	(95% CI)	Rate Ratio	(95% CI)
Descriptive norms	*n* = 239		*n* = 241		*n* = 239	
Age	1.05	(1, 1.11)	1.02	(0.93, 1.12)	1.03	(0.97, 1.09)
Female	0.72 *	(0.53, 0.97)	0.67	(0.39, 1.16)	0.63 **	(0.45, 0.88)
Perceived drinks per week	1.03 **	(1.01, 1.05)	--	--	1.02	(1, 1.04)
Perceived heavy episodic drinking	--	--	15.93 **	(1.99, 127.34)	--	--
Injunctive norms	*n* = 240		*n* = 242		*n* = 241	
Age	1.06 *	(1, 1.11)	1.02	(0.94, 1.12)	1.04	(0.98, 1.1)
Female	0.7 *	(0.51, 0.98)	0.74	(0.43, 1.29)	0.76	(0.53, 1.09)
Perceived approval of alcohol	0.98	(0.85, 1.14)	1.1	(0.87, 1.4)	1.21 *	(1.04, 1.41)

Note: * p < 0.05; ** *p* < 0.01. Student age moderated associations such that relative to students aged 21–25, the association between perceived peer drinks per week (descriptive norms) and personal drinks per week was less pronounced for students aged 26–29 (*β* = −0.07, *p* = 0.025), and the association between greater perceived peer approval of alcohol use (injunctive norms) and personal alcohol-related consequences was less pronounced for students aged 26–29 (*β* = −0.63, *p* = 0.004).

**Table 3:** Rate ratios (and 95% confidence intervals) for associations among past 30-day cannabis use, hours high per week, cannabis-related consequences, and descriptive and injunctive cannabis norms among 2-year college students with recent moderate use.

	Days Using Cannabis		Hours High per Week		Cannabis-Related Consequences	
	Rate Ratio	(95% CI)	Rate Ratio	(95% CI)	Rate Ratio	(95% CI)
Descriptive norms	*n* = 236		*n* = 230		*n* = 227	
Age	0.97	(0.9, 1.04)	1	(0.93, 1.08)	0.99	(0.93, 1.07)
Female	0.8	(0.53, 1.2)	0.81	(0.51, 1.28)	0.52 **	(0.35, 0.79)
Perceived hours high per week	1.01	(0.99, 1.03)	1.02 *	(1, 1.04)	1.01	(0.99, 1.03)
Injunctive norms	*n* = 239		*n* = 231		*n* = 228	
Age	0.96	(0.89, 1.03)	1	(0.93, 1.08)	0.98	(0.92, 1.06)
Female	0.78	(0.52, 1.17)	0.79	(0.5, 1.25)	0.53 **	(0.36, 0.8)
Perceived approval of cannabis	1.15	(0.95, 1.4)	1.13	(0.92, 1.39)	1.09	(0.9, 1.32)

Note: * *p* < 0.05; ** *p* < 0.01. Student age moderated associations such that relative to students aged 21–25, the association between greater perceived peer approval of cannabis use (injunctive norms) and personal cannabis-related consequences was less pronounced for students aged 18–20 (*β* = −0.63, *p* = 0.049).

## Data Availability

The data presented in this study are available upon request from the corresponding author due to privacy restrictions.
